# Dental Department

**Published:** 1900-03

**Authors:** F. S. Casper

**Affiliations:** Austin, Texas


### Dental Department.

F. S. CASPER, D. D. S., AUSTIN, TEXAS.

ARSENICAL ACID IN CEMENTS.—It is, indeed, consoling (?) to the conscientious practitioners of dentistry, who have been constantly using the different brands of cements placed upon the market as a filling material and covering for sensitive dentine and exposed nerves, to read an account of the "Experimental Study of Cements," by J. E. Hinkins, D. D. S., of Chicago, published in the October number of the *Review*.

It has been the prevalent opinion with the profession that in using cement as a filling material, and a covering for sensitive teeth, exposed nerves, etc., they were using a harmless substance which would act as a thorough non-conductor, and protect the teeth from thermal changes, thereby allowing the nerve which had become influenced from exposure, to resume its normal condition. There is now, no doubt, that many cases supposed to have been protected in this manner have returned to the operator with dead or congested nerves. Of course, in such cases, the natural conclusion would be, and doubtless is, that this condition was caused by the amount of exposure before presentation and filling. But now, we can see that if the cement contained the smallest amount of arsenic, the destruction of the pulp was inevitable.

Dr. Hinkins says: "After I had experimented with a number of American cements and found an abundance of arsenic in the oxide of zinc, I thought I had overlooked some important point, so I went to Prof. Long, of the Northwestern University Medical School, who has gone over every cement I have here, and has indorsed my experiments to be correct."

The doctor gives the different tests used, and says: "I should not have been surprised at finding arsenic in the phosphoric acid liquid, as some of the acid of commerce is made by processes which would not exclude arsenic. The investigation of the acid liquids would be a very interesting question, as they seem to vary greatly in composition and behavior. I made no special test in this direction, but noticed this fact incidentally when making the arsenic test."

The doctor also gives the names of several brands of cement which did not contain arsenic. This is some consolation, and we imagine will fall on the paralyzed feeling of victimized dentists like the last lecture of the great yellow fever doctor, Stone, fell upon the ears of the assembled M. D.'s of New York City. In his last lecture on yellow fever, delivered in Bellevue College, New York, Dr. Stone said: "When I first went to New Orleans they (meaning the doctors) were giving 20 grains of calomel at a dose, and they (meaning the patients) sometimes got well."

Of course, if the cements placed upon the markets contain arsenic, instead of protecting the nerves the operator is simply using a destructive agent.

### Dental Items of Interest.

There are a number of different kinds of affliction to which the nerve of teeth are subject, viz.: Irritation, inflammation, congestion, fungus, fatty degeneration, suppuration, calcification, atrophy, putrecence and mummification. To distinguish the difference is a fine point in diagnosis, as the symptoms of several ailments alluded to above are very much alike.

Do not extract temporary or milk teeth too early. Remember that there are only twenty temporary or milk teeth, and the maxilliary bone is just large enough to accommodate that number. In the adult we have thirty-two permanent teeth. If you extract the temporary tooth too early, the maxillary bone will not grow to accommodate thirty-two teeth, consequently you will have abnormal or crowded teeth.

---

CLEANLINESS.—Remember cleanliness is essential to the welfare of temporary teeth. Therefore, the child should be instructed and encouraged to use the brush at as early an age as they are capable of doing so.

---

MUTUAL.—"Well," said the patient, paying his bill, "I shall now go and eat a square meal for the first time in three days." "Same here," quoth the dentist, thrusting the money deep in his pocket.—*Digest.*

